# People with intellectual disabilities and harmful sexual behaviour: Professionals' views on the barriers to prevent harm

**DOI:** 10.1111/jar.13048

**Published:** 2022-11-16

**Authors:** Gøril Brevik Svae, Bjørnar Hassel, Erik Søndenaa

**Affiliations:** ^1^ Department of Neurohabilitation Oslo University Hospital Oslo Norway; ^2^ Division of Clinical Neuroscience, Department of Research and Innovation Oslo University Hospital Oslo Norway; ^3^ Institute of Clinical Medicine University of Oslo Oslo Norway; ^4^ Department of Mental Health Norwegian University of Science and Technology Trondheim Norway; ^5^ St. Olav's Hospital Centre for Research & Education in Forensic Psychiatry Trondheim Norway

**Keywords:** barriers to prevention, criminal justice system, harmful sexual behaviour, intellectual disability, support services

## Abstract

**Background:**

People with intellectual disabilities can be exposed to sexual abuse and they can display harmful sexual behaviour. This study aimed to identify barriers to preventing harmful sexual behaviour in people with intellectual disabilities within the support sector and the justice system.

**Method:**

We conducted focus group interviews with 20 participants from hospital‐based habilitation centres, community residences, schools and the criminal justice system.

**Results:**

The interviews identified a lack of education and guidelines for stakeholders or carers on regulating the sexual behaviour of people with intellectual disabilities. The criminal justice system faces challenges related to prioritising, understanding and communicating. People with intellectual disabilities may lack an understanding of the concepts of sexual consent and acceptable sexual behaviour.

**Conclusion:**

There is a need to improve knowledge about intellectual disability and how to prevent harmful sexual behaviour for professional caregivers in the support sector and the criminal justice system.

## 1 INTRODUCTION

### 1.1 Harmful sexual behaviour and people with intellectual disabilities

Intellectual disability is defined as a reduction in intellectual and adaptive abilities (American Association on Intellectual and Developmental Disabilities, 2021). It can be challenging for people with intellectual disabilities to have healthy sexual lives, and they are less knowledgeable about sexual health compared with the general population (Borawska‐Charko et al., 2016; McDaniels & Fleming, 2016). Some people with intellectual disabilities exhibit harmful sexual behaviour (Malovic et al., 2020) which may be linked to a poor understanding of the concepts of consent and sexual abuse (O'Callaghan & Murphy, 2007). Literature on harmful sexual behaviour is discussed throughout the world, but mainly in Western countries (Medina‐Rico et al., 2017). People with intellectual disabilities can encounter the criminal justice system due to committing sexual offences. When comparing 2220 people with intellectual disabilities and 2085 controls without intellectual disabilities, Nixon et al. (2017) found that people with intellectual disabilities were more likely to have been charged and/or victimised with a sexual or violent offence than those without intellectual disabilities. Contrary to this finding, in a study among the incarcerated, Callahan et al. (2022) found that people with intellectual disabilities were no more likely to commit sexual offences compared to people without this diagnosis. People with intellectual disabilities can sexually abuse others while simultaneously being victims of sexual abuse themselves (Lindsay et al., 2012). Comprised, these studies may indicate that it does not have to be a correlation that people with intellectual disabilities can both be a victim of abuse and offend others. Even so, this can occur in some.

Several studies have examined the prevalence and the risk factors of harmful sexual behaviour among people with intellectual disabilities. A study by Codina and Pereda (2022) found that 35% of the sample had experienced sexual abuse and that the offender was often a known male person and that the victims were likely to be female, to have been declared legally incapable and to have two or more mental health diagnoses. A meta‐analysis by Tomsa et al. (2021) examining the prevalence of sexual abuse among adults with intellectual disabilities found that one in three adults with intellectual disabilities had experienced sexual abuse and that the abuser was most frequently a peer with intellectual disabilities. They found that prevalence was higher among those with mild to severe intellectual disability and low among persons with profound intellectual disability (Tomsa et al., 2021). Recent Norwegian studies have suggested an underreporting of the sexual abuse of people with intellectual disabilities; few cases are investigated (Åker & Johnson, 2020) and there is evidence of poor collaboration between the justice and healthcare sectors in cases involving victims with intellectual disabilities (Åker et al., 2020).

### 1.2 Definition of harmful sexual behaviour

Inspired by Malovic et al. (2020), we have adjusted the definitions of harmful sexual behaviour from Rich (2011) and the National Society for the Prevention of Cruelty to Children (NSPCC, 2017), which consider harmful sexual behaviour to be ‘sexually aggressive, sexually abusive or offensive, or inappropriate sexual behaviours that victimize others’ (Rich, 2011, p. 16). There is a wide range of harmful sexual behaviours, and it is important to consider the individual's age and developmental stage alongside their behaviour (NSPCC, 2017).

### 1.3 Professional caregivers report that harmful sexual behaviour is challenging to handle

How professional caregivers respond to the harmful sexual behaviour of people with intellectual disabilities is an important factor in our research. According to White et al. (2003), being institutionalised tends to be associated with a higher risk of experiencing sexual abuse. They highlight the importance of staff's attitudes, skills and knowledge. In previous research, staff in intellectual disability services have reported that harmful sexual behaviour was challenging for them to handle (Oloidi et al., 2022; Ward et al., 2001). Further, a lack of knowledge among professional caregivers about the sexuality of people with intellectual disabilities has been documented in several studies (Esmail et al., 2010; Lunde, 2014; Lunde et al., 2022; Saxe & Flanagan, 2016; Young et al., 2012). Fyson and Patterson (2020), interviewing staff from residential settings, found variations in staff's understanding of abuse and poor practice. Together, these studies indicate that professional caregivers are uncertain about how they can support people with intellectual disabilities regarding their sexual health. A systematic review by Charitou et al. (2020) exploring how staff support people with intellectual disabilities with relationships and sex found that staff tend to avoid handling sexual issues. Further, this study highlighted that a lack of guidelines and training creates insecurity about how to offer this support (Charitou et al., 2020). Elvegård et al. (2019) addressed the need for an improvements and national (Norwegian) guidelines for supporting vulnerable people at risk of abuse. Strategies to prevent harmful sexual behaviour among people with intellectual disabilities must account for unique challenges both at the individual level of people with intellectual disabilities and at the societal level, including the institutions responsible for their care (Nixon et al., 2017).

### 1.4 The situation in Norway

The present study took place in Norway, where people with intellectual disabilities largely live in communal residences (Tøssebro, 2016). There are no institutions that specifically serve those who display sexually offensive behaviours. When the communal residences are unable to provide sufficient support in situations of severe mental health issues or criminal acts, specialist mental health services and correctional services are made responsible for care and treatment. Norwegian legislation requires that all use of physical restraint be formally registered and thoroughly documented, including any form of physical restraint applied in interventions intended to prevent or respond to incidents of harmful sexual behaviour. The intention of the legislation is to prevent harmful behaviour while still minimising the use of restraints in the care of people with intellectual disabilities (Helse‐og omsorgstjenesteloven, 2017, §9‐1–§9‐14). According to The Norwegian Law on Health and Care Services, health care staff have the duty to intervene if a patient or client may be in danger (Helsepersonelloven, 2017, §17). At the same time, health care staff have a duty of secrecy (Helsepersonelloven, 2008, §21) which involves preventing others from becoming aware of confidential information.

People with intellectual disabilities receive help and guidance from support services, including communal residences, schools, day care centres, facilitated work environments, and, in cases of harmful sexual behaviour, the criminal justice system. Barnahus is a part of the justice sector which protects the legal rights of minors and people with intellectual disabilities (Statens Barnehus, 2021). The main objectives of Barnahus are to facilitate the legal process and to provide alleged victims or perpetrators with support and treatment (Bakketeig, 2017). Barnahus is mandated to improve the legal rights of both victims and alleged offenders with intellectual disabilities. However, little is known about how these institutions prevent harmful sexual behaviour among people with intellectual disabilities.

This study focuses on people with intellectual disabilities of all ages. We aimed to explore the experiences of participants within different sectors of the care, support and justice systems who had extensive experience with harmful sexual behaviour in people with intellectual disabilities. From these experiences, which factors within the support and justice systems can we identify as potentially relevant to the prevention of harmful sexual behaviour?

## 2 METHODS

### 2.1 Recruitment of participants and data collection

The study was initiated from a hospital‐based habilitation centre in Oslo, Norway. We followed the recommendations of Oslo University Hospital's Data Protection Officer at Oslo University Hospital for processing personal data, case number 19/03029. Written informed consent was obtained from all included participants.

Focus group interview nr. 1 was conducted in a city in central Norway and focus group interview nr. 2 in south‐east Norway. Snowball selection was used to recruit participants from the author's network (Braun & Clarke, 2013). Participants were strategically selected from both rural and urban areas. The eligibility criteria were that participants had extensive experience with harmful sexual behaviour in people with intellectual disabilities. All participants were recruited because we thought their positions in the field would provide the experience necessary to contribute appropriate information to the study. Many of the participants were recruited from a national network of professionals from hospital‐based habilitation centres who work with people with intellectual disabilities and sexual health (NFSS, 2020). Their professional positions were all within the health, social, care and criminal justice services (Table 1).

**TABLE 1:** Demographic characteristics of participants (*N* = 20)

Gender	
Female participant	15
Male participant	5
Representing urban areas	14
Representing rural areas	6
Work sector	
The Barnahus/police	5
The criminal justice system	3
Specialist hospital‐based habilitation centres	6
Special schools	2
Community caring services	4

Potential participants (*n* = 30) received written information in e‐mails inviting them to take part in the study, followed up with a phone call or email a week or two later. Ten persons declined to participate due to time constraints or because they felt they lacked sufficient experience. We conducted two focus group interviews, one with 8 participants, and another with 12 participants. The participants were 5 men and 15 women, 14 participants represented urban areas and 6 represented rural areas. The participants had the following backgrounds: psychologist, teacher, social educator, nurse, child welfare worker, police officer, and lawyer. The participants from the police represented different departments of the criminal justice system, working with facilitated interrogations, the court, and the correctional services. The other participants were from specialist hospital‐based habilitation centres, special schools and community care services.

### 2.2 Focus group interviews

Focus groups were chosen because they are considered to be a useful method when the purpose of the research is to study group norms, processes, interactions and behaviours (Barbour & Flick, 2007). A semi‐structured interview guide was developed by testing a pilot version on three experienced specialists from the hospital‐based habilitation centre in Oslo. The focus group interviews were conducted in August 2019. The interviews focused on the participants' experiences of, thoughts and attitudes regarding harmful sexual behaviour among people with intellectual disabilities. A semi‐structured interview guide was used, with open‐ended questions intended to spark discussion, for example, ‘What have you experienced to be the key to successful prevention of harmful sexual behaviour in people with intellectual disability?’ They then moved on to more specific questions about experiences with support systems, such as: ‘What obstacles have you experienced as barriers to the prevention of harmful sexual behaviour in people with intellectual disabilities in support systems such as communal residences, schools and day care centres?’ Finally, we asked about the participants' suggestions for improvements (Table 2). The first author conducted the interviews, while another author took notes. The interviews were audiotaped and lasted approximately 90 min. The data were managed in line with Oslo University Hospital guidelines. The data were stored in a secure area and deleted when transcription was completed.

**TABLE 2:** Interview guide for focus group interview: People with intellectual disabilities and harmful sexual behaviour

(1) Introduce myself (first author)
(a) Introduction to the project and the purpose of the focus group interview. A short overview of the theme and potential questions.
(b) The focus group interview is an excellent method for sharing experiences of success factors and barriers. Please, do not talk to me; speak with each other. My job is to steer the conversation, and, to begin with, it would be nice if we could start by talking about the topic more generally to capture any spontaneous thoughts and opinions.
(c) Give information about the tape recorder and explain that notes will be taken if they are necessary to keep the conversation on track.
(d) Review information about consenting to participation, confidentiality and anonymity.
(2) Time to talk about the topic more generally (30–45 min)
(3) Questions
(a) What kind of experience do you have with the subject? What is your role?
(b) What have you experienced to be the key to successful prevention of harmful sexual behaviour in people with intellectual disabilities?
(c) Are there any factors related to the various target persons (people with intellectual disabilities and harmful sexual behaviour) that have contributed to success? Factors that can be elaborated on? Which factors can be barriers?
(d) Do you have any experience with the staff who support them (people with intellectual disabilities and harmful sexual behaviour)? What aspects of the follow‐up made it go well? What, if anything, has been challenging?
(e) What thoughts do you have about the staff's competence and knowledge regarding harmful sexual behaviour?
(f) What obstacles have you experienced as barriers to the prevention of harmful sexual behaviour in people with intellectual disabilities in support systems such as communal residences, schools and day care centres?
(g) What is your experience with the national legal regulation of applying restraint measures (chapter 9) in this context?
(h) What experience do you have with the families of people with intellectual disabilities and harmful sexual behaviour? Positive and negative aspects?
(i) Now that we have discussed your experiences; what measures do you think can be taken to contribute to good health services for the target group?

### 2.3 Analysis

Data underwent thematic analysis according to the guidelines described by Braun and Clarke (2006). In the first phases, the first author transcribed the audio‐interviews with pseudonyms connected to each participant. Microsoft Word, was used to work with the dataset, so no additional software was required for the qualitative analyses. The first and the second authors also read through the transcriptions several times to become familiar with the data. The first and second authors sorted the text by general themes using initial codes. The researchers discussed the material until an agreement was reached on the coding structures based on patterns within the data. The themes were sorted for thematic analysis, and the third author sent reports by email in which the themes were discussed. During the last phase, the themes were reviewed between the transcribed material and the themes and subthemes. All authors were involved in this process which resulted in two themes being merged. The quotes in the article are translated from Norwegian to English, and have been reviewed to ensure that their original meaning has not been lost or altered.

## 3 RESULTS

The thematic analysis resulted in two themes and nine subthemes, which are presented below. We found that the two themes were system‐ and profession specific. One theme was about barriers in the support system, and the other about barriers in the services within, and related to, the criminal justice system.

### 3.1 Barriers for prevention of harmful sexual behaviour within the support system

The participants described barriers within the support system that obstruct the prevention of harmful sexual behaviour. Keywords for these barriers were ‘need for knowledge’, ‘missing professional support’ and ‘low priority’.

#### 3.1.1 The need for sexual knowledge among people with intellectual disabilities

The majority of the participants said that many people with intellectual disabilities have little knowledge about sexuality, and emphasised the importance of sex education to prevent harmful sexual behaviour. Many of the participants highlighted the need for improved sexual education for people with intellectual disabilities. Some commented that sexual education tended to be too general and not sufficiently customised to the needs of individuals with intellectual disabilities. One participant experienced that some people who had been incarcerated had difficulties handling sexual arousal as a part of their emotions, and discovered they had never learned anything about it. She said:

Feelings as a theme is something I spend a lot of time on with them, to teach them what happiness, fear, sadness and sexual arousal are. When talking about emotions in general, sexual arousal is often excluded. It's a subject that's difficult to talk about. Handling sexual arousal is a part of sexuality, to get help to regulate it. (P2, focus group 1).

The participants described how people with intellectual disabilities often lack the words to describe sexual emotions or events because of limited education on sexual health. Many of the participants reported that education in sexual health is reliant on individual initiatives and is not encouraged by the support system as a whole. Some participants described how the transference between the services for children and adults functions as a barrier. One participant said that people with intellectual disabilities do not get much sex education as long as they are considered to be children (up to the age of 18), but that it may be too late to begin this education at 18, when they are considered to be adults, because sexual behaviour is often established at an earlier age.

The participants indicated a need for people with intellectual disabilities to be taught sexual codes and limits. One participant working at a special school explained that sexualised behaviour was the reason they started providing sex education. She said:

We started sex education because we saw how they behaved and “did it” in the laundry room and everywhere, and we were wondering if they had had any education about sex, because they were having several boyfriends and girlfriends, but they were “allowed.” (P 14, focus group 2).

The participants believe that a lot of the sexualised behaviour in people with intellectual disabilities is inappropriate because they have not had any sex education. For instance, one participant said there is a thin line between acceptable and unacceptable behaviour online, and that young people with intellectual disabilities need more knowledge on this subject. Another participant pointed out that it complicates police investigations if people with intellectual disabilities have difficulties explaining themselves on the topic of sexual abuse.

#### 3.1.2 Lack of professional support for people with intellectual disabilities and harmful sexual behaviour

The participants expressed concerns about the lack of follow‐up from the support system for people with intellectual disabilities and harmful sexual behaviour. They believed that much harmful sexual behaviour could be prevented by the provision of guidance and professional support at an earlier age. One participant from a specialist hospital‐based habilitation centre said: ‘Many need frequent repetitions, frequent follow‐up, they need someone they know to talk to, but they don't have that’ (P 5, focus group 1).

Participants from both focus groups said that a common challenge is that some people with intellectual disabilities do not cooperate and do not agree that they need help. Many of the participants had experienced the difficulty of trying to give support to people with intellectual disabilities who do not want help.

Several participants highlighted the need for increased competence among staff in the support system, because harmful sexual behaviour is so challenging to handle. One professional pointed out that the lack of knowledge in the support system is connected to the high turnover of staff – when experienced caregivers leave these services, their competence also disappears. A participant from a specialist hospital‐based habilitation centre said: ‘There are committed caregivers with competence in the support system, but they get burned out and find new jobs. The work around the targeted person with harmful sexual behaviour reset to zero, and things go wrong again’ (P 8, focus group 1).

The professionals shared several cases where people with intellectual disabilities and severely harmful sexual behaviour had been without professional support for years despite their obvious needs. Several participants had experienced cases where people with intellectual disabilities had begun harmful sexual behaviour after losing their professional support services. It was pointed out that promoting and facilitating sexual health for people with intellectual disabilities is continuous work, often required throughout their lives. Further, the professionals often experienced that the local support system refrained from intervening because they found it ethically challenging.

Several of the professionals said that the support services neglected sexual health. Participant 5 in focus group 1 said: ‘I have tried to get sexual health into individual support plans for almost 20 years, but there is no interest for it’, meaning that the support system did not follow up on this initiative.

Another participant wanted more collaboration between the specialist hospital‐based centres for children and adults so that people with intellectual disabilities could receive sexual health interventions earlier. In both focus groups the participants pointed out the importance of the support system management acknowledging the necessity of their work with sexual health.

#### 3.1.3 Neglect of the duty to intervene to prevent harmful behaviour

In both focus groups the participants highlighted the importance of the ‘duty to intervene to prevent harmful sexual behaviour’. However, most believed that health care staff neglect their duty to stop abuse. Professionals from the criminal justice system said that people with intellectual disabilities are often represented on both sides, having been sexually abused as well as having sexually abused others. Several participants knew of cases where staff in the support system were aware of sexual abuse but did not intervene. Many of the professionals felt that the sexuality of people with intellectual disabilities was too private and delicate a matter to be handled by the support staff. One participant from the specialist hospital‐based habilitation centre shared her experience:

We have some cases where harmful sexual behaviour continues, the caregivers don't know what to do, and the targeted person rejects support. We have guided the staff into a position to give the targeted person more help. Then they discover that there are significant problems with hygiene, knowledge about boundaries and more extensive functional needs (P 16, focus group 2)

Several of the participants described being contacted by support staff who wanted help and guidance on how to handle cases of sexual abuse which even the professionals themselves found challenging. One participant pointed out that there has been a long tradition in Norway of not sharing information because of the strict confidentiality rules in the health care system which has stopped support staff from seeking professional help to prevent sexual abuse.

### 3.2 Barriers to prevention of harmful sexual behaviour in the criminal justice system

The participants in the study described various barriers to the prevention of harmful sexual behaviour that are specific to the criminal justice system and child welfare system, including the police, Barnahus, courts and prisons. Many participants stated that there are alternatives to the criminal justice system, but that these options are not used consistently.

#### 3.2.1 Children with intellectual disabilities are being treated differently from typically developing children

When a child (below age 18) commits harmful sexual behaviour, the police can offer facilitated interview in a Barnahus especially designed for this purpose. A participant from the Barnahus/police described the discriminative practices of the child welfare system:

The police notify the child welfare system when there is a facilitated interview, but I have never experienced them showing up. They don't come if it is about a child with a disability, and they have a lot of experience and can give support in a crisis like a facilitated interview (P 17, focus group 2).

When the child welfare system does respond in a case involving a child with an intellectual disability, it is because a sibling without an intellectual disability is involved too. A participant from a specialist hospital‐based habilitation centre echoed this statement by saying: ‘Yes, that is our experience too’ She continued: ‘So, you have to argue every time you are in contact with the child welfare system that this is a case for them (P 11, focus group 2)’.

#### 3.2.2 Undiagnosed intellectual disability

Many participants worried that people who had been diagnosed with intellectual disabilities as adults had missed out on customised education about proper sexual behaviour. Several participants knew about people who had been suspected of sexual abuse, and who had received an intellectual disability diagnosis as adults. One participant from the Barnahus/police had been worried about a young man who was suspected of sexual abuse. The participant believed this man needed help for his harmful sexual behaviour, but he refused to consent:

I wanted him examined, we needed a way in and to connect the [help] system to him, so we managed to do that. They said that the young man has been attending special school all his life, is illiterate, and we discovered that he cannot write his name (P 18, focus group 2).

This example illustrates how intellectual disabilities can go unrecognised in the criminal justice system due to lack of consent. Other participants commented that it may be problematic to get a diagnosis of intellectual disability after sentencing, when the conviction was based on an incomplete knowledge of the person on trial.

#### 3.2.3 Delays in the criminal justice system

The participants discussed the problematic aspects of the lengthy time it takes for the police to investigate harmful sexual behaviour committed by people with intellectual disabilities. A participant said that the justice system is adapted for people without disabilities, and it is a particular challenge for those who have an intellectual disability as a diagnosis. Many individuals are interrogated when they are caught exhibiting harmful sexual behaviour for the first time, but when nothing else happens, they reoffend because they do not understand what they did wrong. One participant from the Barnahus/police expressed a lack of competence in the justice system:

They are caught for the first time and go to interrogations, the case is ongoing but they see that nothing is happening, thing takes time. It is a lack of competence. We do not have a system to follow them up, and you do not know how to do it (P 19, focus group 2).

Other participants said that these cases do not seem to be a priority.

#### 3.2.4 Long‐term follow‐up from the criminal justice system

In one of the focus groups, the participants discussed the lack of long‐term follow‐up of people with intellectual disabilities with harmful sexual behaviour. However, one participant from the criminal justice system said that a person who is convicted of sexual offences and is considered to be at risk of reoffending will remain incarcerated and continue to receive professional support. Several participants commented that a person with an intellectual disability would have better access to professional care in prison compared to with the community support services. One participant (P 2) from the criminal justice system in focus group 1 questioned the ethics of the situation: ‘Should they remain incarcerated to receive the care and support they need due to their intellectual disability?’ Some participants found this contrast interesting, those who are considered at risk of reoffending are provided with support, yet, outside of the criminal justice system, people who could make behavioural changes do not receive the support they need.

#### 3.2.5 Lack of support after release from custody or discharge from incarceration

In one of the focus groups, many of the participants had experienced that the local support systems for people with intellectual disabilities in several cities had refused to follow up after an individual was released from custody or discharged after incarceration. One participant said that the service administration often ignores these people, saying that they have no services to offer them. Other participants also mentioned this, and said that there are laws that can be applied to people with intellectual disabilities and harmful sexual behaviour. One participant from a specialist hospital‐based habilitation centre continued:

It's about how you write and talk about it. It's quite correct that you can't use chapter 9 [National legal regulation of applying restraint measures] to prevent crime. Still, you can use chapter 9 to prevent a person from harming themselves or others or to prevent property damage (P 5, focus group 1).

A professional from another specialist hospital‐based habilitation centre agreed and commented that, depending on how the case is presented: ‘Chapter 9 can be used to help a person with intellectual disabilities avoid committing harmful sexual behaviour and thereby avoid conviction within the criminal justice system’ (P 8, focus group 1). The participants maintained that, if they knew about it, the local support systems could use *chapter 9* to efficiently prevent harmful sexual behaviour. The proper use of legal restraint measures may help prevent harmful sexual behaviour, but these regulations are not applied consistently across the country.

## 4 DISCUSSION

In this study, we identified several barriers to the successful prevention of harmful sexual behaviour in people with intellectual disabilities. Support systems need more knowledge about sexual abuse among people with intellectual disabilities and guidelines on how to intervene to avoid harmful sexual behaviour. In the criminal justice system there is a need for more knowledge about intellectual disabilities as a lack of understanding about the diagnosis creates inequality in the treatment of children with intellectual disabilities, as well as a lack of procedures for identifying people with intellectual disabilities and possible alternatives to avoid sentencing.

### 4.1 A lack of knowledge about the sexual health of people with intellectual disabilities

Overall, the professionals highlighted the need for people with intellectual disabilities to improve their sexual knowledge. This finding is in line with a systematic review (Brown & McCann, 2018) which shows that people with intellectual disabilities themselves want more sex education. Therefore, the present study points to the need for improved sexual education for people with intellectual disabilities. Gougeon (2009) has argued that the sexual education provided for this group is unspecific and has not been adapted to reflect the reality of their lives. Others believe that there is a lack of conversation about pleasure and positive sexual feelings and that this subject is not prioritised by the professionals who speak with people with intellectual disabilities (Alexander & Taylor Gomez, 2017).

This study suggests that the importance of preventing harmful sexual behaviour has not been recognised within the support services for people with intellectual disabilities. These systems have only limited resources, and no guidelines, for the prevention of such behaviour. In addition, support staff appear to be stuck in a conflict between individual self‐determination for people with intellectual disabilities and the need to protect them from abuse. This conclusion is in line with other studies in the field (Eastgate et al., 2012; Wickström et al., 2020). The present study highlights the lack of knowledge about intellectual disabilities and sexual health among support staff. It is important to ensure a good understanding of sexual health, and several studies show that educational interventions with support staff yield positive results (Grieve et al., 2009; Meaney‐Tavares & Gavidia‐Payne, 2012; Pebdani, 2016).

More discussion about the regulation of the sexual rights of people with intellectual disabilities is needed. Our findings show the need for guidelines for professional staff on how to support the sexual lives of people with intellectual disabilities. The results of this study should encourage policy and practice intended to strengthen sexual education for staff and people with intellectual disabilities, thereby preventing harmful sexual behaviour. Our study had only four participants representing the community care services and barriers in the support system are an essential issue for future research. Further studies are needed to explore the experiences of managers and professional decision‐makers in support systems.

### 4.2 Criminal justice system inadequacy

This study reveals the challenges that people with intellectual disabilities and harmful sexual behaviour face in the criminal justice system in relation to other services like the child welfare system, the police, Barnahus, courts and prisons. The present study shows that there is a need for greater competence regarding intellectual disabilities among those who work in these services.

The professionals believed that children with intellectual disabilities do not have the same access to assistance from the child welfare system as children without disabilities. These results are consistent with a study (Cooke & Standen, 2002) which found that social workers were worried that there was a tendency to overlook the abuse of children with disabilities. This situation can be a result of a lack of competence concerning children with disabilities within the child welfare system. A lack of competence about disability can lead to discriminatory practices that result in children with intellectual disabilities not getting the same help as children with no diagnosis.

Further, people with intellectual disabilities are frequently not identified as such in the criminal justice system (Søndenaa et al., 2008), meaning that their needs for adapted care and treatment may go unrecognised. The present study indicates that some people with intellectual disabilities and harmful sexual behaviour can be considered to have a life‐long risk of reoffending. This finding is in line with a previous study which identified a pattern of frequent reoffending among some people within this group (Søndenaa et al., 2008) as well as significant problems after discharge from prison, including great difficulty reintegrating into society (Murphy et al., 2017). Social reintegration after release from prison is made difficult by the lack of cooperation between the criminal justice system and community services. This disconnect, which has been documented previously (Chiu et al., 2020), was illustrated in the present study by the fragmentation of services for past offenders with intellectual disabilities. On the other hand, there is a growing understanding that people with intellectual disabilities are often victims (Nixon et al., 2017; Petersilia, 2001). To prevent further victimisation, knowledge within the legal system is crucial for identifying people with intellectual disabilities and providing them with the correct help and treatment. Based on the findings of this study, we suggest some potential implications. Targeted interventions could strengthen knowledge about intellectual disabilities and harmful sexual behaviour in the criminal justice and child welfare systems. The higher education system should address intellectual disabilities and harmful sexual behaviour courses for future professionals to ensure they will be able to meet their prospective clients' needs. More research is needed concerning children with intellectual disabilities and potentially discriminatory practices in the child welfare system.

## 5 STRENGTHS AND LIMITATIONS

A strength of the present study is the varied background of the participants, comprising professional caregivers in community residences, special schools, specialists at hospital‐based habilitation centres, and representatives of the criminal justice system. This variety should ensure the identification of several barriers to the prevention of harmful sexual behaviour in people with intellectual disabilities. Although the qualitative results cannot be generalised, some of these barriers may be transferable to other countries with similar situations. Different cultures have different approaches to issues of sexuality in people with intellectual disabilities (Medina‐Rico et al., 2017). Barriers of the support system on the competency of handling harmful sexual behaviour, and barriers of the criminal justice services on competency concerning intellectual disabilities, are possibly relevant to other countries.

Another strength may be the clinical background of the interviewer (GBS) who, working in the same field, may have influenced the nature of the interviews. This study has some weaknesses. The participants were not asked about background information such as age and length of employment, and male participants were underrepresented in the material. To ensure the trustworthiness of the study, the process of data collection is described in detail. A strength is that the interview guide was pilot tested beforehand by three experienced specialists from specialist hospital‐based habilitation centres.

There is a need for further studies to identify barriers to successful prevention of harmful sexual behaviour in people with intellectual disabilities. Such studies should focus on mechanisms behind the shortcomings of support services in the community, the child welfare system and the criminal justice system. We believe that this study highlights the need for better policies and guidelines regarding the sexual health of people with intellectual disabilities.

## 6 CONCLUSION

This study highlighted several barriers to the prevention of harmful sexual behaviour among people with intellectual disabilities. Behind these barriers is a lack of knowledge about the sexual health of people with intellectual disabilities. Guidelines on how to intervene and prevent harmful sexual behaviour in people with intellectual disabilities are lacking within the community support services, the child welfare services and the criminal justice system. Future studies should address the mechanisms behind the shortcomings of community support services, the child welfare services and the criminal justice system.

## CONFLICT OF INTEREST

All authors declare that they have no conflicts of interest in relation to the submitted work.

## Data Availability

Data may be available from the corresponding author on reasonable request and following ethical approval.
